# Patient with pontine warning syndrome and bilateral posterior internuclear ophthalmoplegia: case report

**DOI:** 10.1186/1471-2377-10-55

**Published:** 2010-06-28

**Authors:** Hai Wei Huang, Shen Wen He, Shuang Quan Tan, Li Li Su

**Affiliations:** 1 Department of Neurology and Stroke Center, the First Affiliated Hospital, Sun Yat-Sen University, No. 58 Zhongshan Road 2, Guangzhou, 510080, PR China

## Abstract

**Background:**

Capsular warning syndrome was first described in 1993, featured with repetitive episodes of motor and/or sensory dysfunction without cortical signs. Recently, it has been demonstrated that clinically typical capsular warning syndrome can be associated with pontine infarct and the term â€œpontine warning syndromeâ€� was coined.

**Case Presentation:**

A 54-year-old woman with a history of hypertension was seen with profound left-sided hemiplegia. She had had 3 episodes of left-sided weakness before complete hemiplegia. Her speech was slurred. Left central facial palsy and hemiglossoplegia were presented. Her left plantar response was extensor and bilateral posterior internuclear ophthalmoplegia was seen on neurologic examination. Biochemical tests revealed hyperglycemia and dyslipidemia on the next day. MRI demonstrated an acute right paramedian pontine infarct. The patient was commenced on oral clopidogrel, atorvastatin and acarbose. After 23 days of hospitalization, she was discharged with severe left hemiplegia.

**Conclusions:**

1) Pontine warning syndrome may be underestimated and understudied. 2) Posterior internuclear ophthalmoplegia is a rare clinical sign in cerebrovascular diseases, while it can help to locate a brainstem lesion rather than an internal capsular one. 3) Blood pressure lowing administration may be improper for patients with pontine warning syndrome.

## Background

Capsular warning syndrome was first described by Donnan et al, denotes a burst of stereotyped transient ischemic attacks (TIAs), clustered within a brief period of time, with motor and/or sensory dysfunction and no cortical signs, and associated with a high risk of internal capsule infarction [1,2]. However, clinically typical capsular warning syndrome can be linked to a pontine infarct [3-6]. Recently Saposnik and Caplan *et al *coined the term "Pontine warning syndrome" to characterize recurrent stereotyped episodes resembling those of capsular warning syndrome associated with a high risk of imminent basilar artery branch infarction and a permanent deficit [7]. In this article, we reported a case of a 54-year-old woman admitted to our stroke center a few months ago, who experienced three stereotyped episodes of left-sided hemiplegia within 7 hours and developed an acute pontine infarct eventually.

## Case Presentation

**Figure 1:**
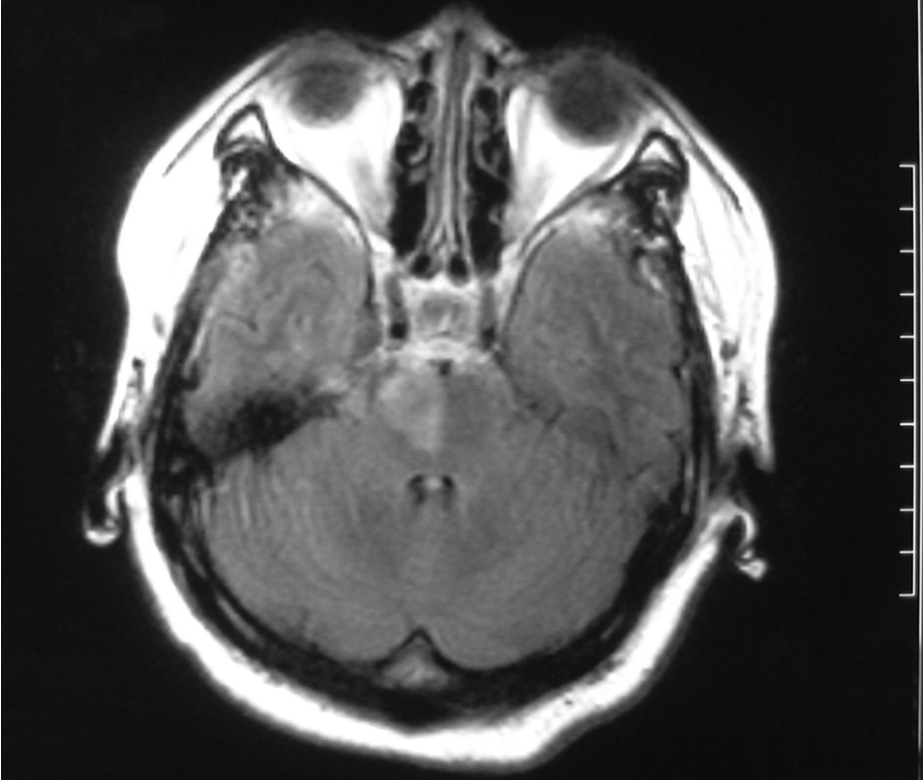
**Magnetic resonance imaging performed 6 days after onset of the fixed hemiplegia**. Axial fluid-attenuated inversion recovery (FLAIR) image shows well-delineated increased signal in the right aspect of the paramedian pontine.

A 54-year-old woman experienced an episode of left-sided hemiplegia at 5:30 AM, March 29, 2009, when she was about to get up from bed. The initial episode lasted 5 minutes and disappeared spontaneously. Two subsequent episodes of the stereotype recurred and lasted 20 minutes and 30 minutes respectively, with complete recovery between such that she could walk, eat and go to the bathroom by herself. At 1 PM she developed severe left-sided hemiplegia and moderate dysarthria, which lasted 6 hours without recovery. Then she was sent to our emergency department in a wheel chair. Her general examination revealed a blood pressure of 120/75 mm Hg, and other findings were unremarkable. On neurologic examination, she was alert with moderate dysarthria, presenting with severe left central facial palsy and mild left hemiglossoplegia. Left-sided hemiplegia was severe in the upper and lower limbs (Medical Research Council scale grade 0), with decreased tone and left extensor plantar response. No sensory or cortical signs were seen. Her neurologic status was 10 on the National Institutes of Health Stroke Scale (NIHSS). She had had a 10-year history of hypertension, and started on oral nifedipine 10 mg three times per day in the last two years. She had seldom measured her blood pressure levels so that she did not know the exact value of her blood pressure. Her serum low density lipoprotein-cholesterol concentration was 3.83 mmol/L. Fasting blood glucose, 30-minutes blood glucose and 2-hours blood glucose after oral glucose tolerance test were 5.80 mmol/L, 14.08 mmol/L and 12.80 mmol/L, respectively. No significant abnormality was detected on cranial CT scan on admission. MRI revealed an acute right paramedian pontine infarct 6 days after the onset of the fixed hemiplegia (Figure1). MRA showed diffused mild atherosclerosis, and no significant stenosis or occlusion within major intracranial arteries. She was commenced on oral clopidogrel, atorvastatin and acarbose. After 23 days of hospitalization, she was discharged with severe left hemiplegia (grade 3 in the proximal and grade 1 in the distal of the left limbs), and her neurologic status was 5 on NIHSS.

## Conclusions

The vascular risk factors in our patient included hypertension, diabetes mellitus and dyslipidemia, in concordance with previously published reports [2,3,5-8]. As a consequence of these risk factors, intracranial artery stenosis or occlusion was speculated to be the main pathological basis for those "warning syndrome" by most authors [1,2,6,7]. The atheroma in a large intracranial artery may occlude the origin of a penetrating branch and lead to hypoperfusion, which was demonstrated to be a major mechanism of ischemic stroke [9]. It has been demonstrated a high prevalence of intracranial artery stenosis among Asian, Black and Hispanic population [10,11]. Furthermore, in a population-based study in southern China, we found 30 person with asymptomatic basilar artery stenosis among 1005 participants with transcranial Doppler, demonstrating a prevalence of 2.90% (data unpublished), considerably higher compared to that of 1.41% in patients from United Kingdom with ischemic stroke of non-POCI (posterior circulation infarction) subtype reported by Mead *et al *[12]. Accordingly, a recent article by some colleagues from our stroke center reported pontine infarction accounted for up to 10.2% of the first-ever ischemic stroke [13]. Given to the relatively poor clinical outcome, pontine warning syndrome may be underestimated and understudied, especially in populations with high prevalence of intracranial artery stenosis.

These "warning syndromes" are characterized by the identifiably stereotyped and crescendo nature of repetitive ischemic episodes. Although the hypothesis has not been verified in any experimental models, it is widely accepted that the repetitive episodes of neurologic deficit were associated with intermittent hypoperfusion of the vascular territory of terminal arteries with insufficient collateral flow, due to penetrating branch disease rather than lipohyalinosis. An anecdotal report may provide further evidence for this hypothesis. Six patients presented with intermittently decreased blood pressure, coinciding with clinical worsening and leading to definite stroke in 4 of them. One patient received noradrenalin, which allowed stabilization of the blood pressure values and complete resolution of the neurological symptoms [14]. Our patient had a history of nifedipine-treated hypertension, and the blood pressure level was "normal" on admission. We presumed lowered blood pressure may involve in the pathogenesis of her pontine infarct. Although recently some reports showed that blood pressure lowing in acute stage might favor the clinical outcome of ischemic stroke [15], blood pressure lowing administration may be improper for patients with pontine warning syndrome.

The 64-year-old patient by Saposnik *et al *had had 2 episodes of complete bilateral horizontal conjugate gaze palsy with unimpaired consciousness lasting for 5 minutes each [7]. Similarly, our patient demonstrated bilateral posterior internuclear ophthalmoplegia on admission. Bilateral abduction palsy were seen when she tried to remain horizontal lateral gaze-holding. However, the rim of the corneal could transiently reach the outer canthus in voluntary saccades, pursuit movements or vestibulo-ocular responses. In general, posterior internuclear ophthalmoplegia is caused by lesions interrupting the connection between the paramedian pontine reticular formation (PPRF) and the ipsilateral abducens nucleus. A patient with multiple myeloma who developed left posterior internuclear ophthalmoplegia was reported by Bogousslavsky *et al *[16]. At autopsy multiple small infarcts were found in the left PPRF extending towards the abducens nucleus, which was involved only in its inferior pole [16]. Posterior internuclear ophthalmoplegia is a clinically rare sign in cerebrovascular disease and may be of great value indicating a pontine lesion rather than other possible locations that could also associate with motor and/or sensory dysfunction. However, the mechanism that a unilateral pontine infarct could cause such a symmetric sign of bilateral posterior internuclear ophthalmoplegia should be further clarified.

Capsular warning syndrome had been proposed to be ideal to formally test various therapies because of the clinically well recognized features and the relatively high incidence of subsequent ischemic stroke [2]. However, this form of TIA seems to be resistant to classical therapies, including anticoagulation, aspirin and hemodilution [2,6]. A combination of aspirin and clopidogrel of loading dose was reported to be effective in 2 cases of capsular warning syndrome [8], but further evidence from randomized controlled trials has not been found to date. Intravenous thrombosis may be a promising therapy for preventing the permanent deficit [17,18]. Unfortunately, the nature of dramatical recovery may impede some patients arriving at hospital within therapeutic window, especially in developing countries. Our patient took for granted that the episode of neurological deficit might resolve spontaneously, such that she chose to wait and stay at home until 6 hours passed. Therefore, propagation that "TIA is an emergency requiring urgent interventions [19]" should take an important part in public heath education.

## Consent

Written informed consent was obtained from the patient for publication of this case report and accompanying images. A copy of the written consent is available for review by the Editor-in-Chief of this journal.

## Competing interests

The authors declare that they have no competing interests.

## Authors' contributions

All authors stated above made substantive intellectual contributions to a published case report. SWH treated the patient and drafted the manuscript. HWH treated the patient and revised the manuscript. SQT helped to draft the manuscript. LLS helped to draft the manuscript. And all authors read and approved the final manuscript.

## Pre-publication history

The pre-publication history for this paper can be accessed here:

http://www.biomedcentral.com/1471-2377/10/55/prepub
